# Calomel as a Remedy in Diseases of the Eye

**Published:** 1844-11

**Authors:** 


*Calomel as a Remedy in Diseases of the Eye.*—On the recommendation of Dr. Major, of Lausanne, Dr. Fricke frequently employs calomel, in the form of powder, with surprising success, where other remedies have failed to produce any effect. The diseases in which he uses it are chiefly: scrofulous rheumatic, and catarrhal opthalmia, particularly when combined with great structural alteration of the conjunctiva and iris, or with effusion into the anterior chamber, and deposition on the cornea. Violent inflammation arose from its use in but two patients, who had formerly taken iodide of potassium for some syphilitic affection. The calomel turned of a yellowish green color in these cases, and this led Dr. Fricke to believe that the iodide of potassium, contained in the lachrymal secretion, combined with the mercury, so as to form a violent stimulant. The author used calomel in all the cases recommended by Fricke, and never observed any other than good effects. He generally blows the powder into the eye by means of a quill, and considers this method less irritating to the organ than the usual manner. He generally takes equal parts of calomel and fine sugar. As to the question, whether its action is mechanical or dynamic—we must certainly decide in favor of the latter; otherwise, every indifferent powder would have the same effect.

*Dr. Neuber*, from *Schmidts Jahrbucher:* from *Ibid.*

